# Adding an anaerobic step can rapidly inhibit sludge bulking in SBR reactor

**DOI:** 10.1038/s41598-019-47304-3

**Published:** 2019-07-26

**Authors:** Junqin Yao, Jiaqi Liu, Yanjiang Zhang, Shuang Xu, Ying Hong, Yinguang Chen

**Affiliations:** 1 College of Resources and Environmental Science, Xinjiang University, Urumqi, 830046 China; 2 College of Environmental and Energy Engineering, Tongji University, Shanghai, 200092 China

**Keywords:** Environmental impact, Water microbiology

## Abstract

Activated sludge from wastewater treatment plants was seeded into a sequencing batch reactor (SBR) in which synthetic wastewater was used as the influent. The sludge was bulked by decreasing the concentration of dissolved oxygen (DO). By adding a 30 min step of anaerobic stirring after the water inflow, the sludge bulking was rapidly inhibited after 10 running cycles, and the sludge volume index (SVI) decreased from 222 to 74 mL·g^−1^. The results of high-throughput sequencing showed that the relative abundance of bacteria *Thiothrix*, bacteria *norank_o*_*Sphingobacteriales* and fungi *Trichosporon* was increased by 6.3, 4.3 and 81.2%, after initial SBR stages, but these bacteria were inhibited by the addition of an anaerobic step, as their relative abundances decreased by 0.7, 0.8 and 14.7%, respectively. The proliferation of *Thiothrix*, *norank_o_Sphingobacteriales* and *Trichosporon* was the primary reason for the observed sludge bulking in the reactor. After the anaerobic step was added, the sludge extracellular polymeric substances (EPS) concentration was increased from 84.4 to 104.0 mg·(gMLSS)^−1^ (grams of mixed liquor suspended solids). Thus, the addition of an anaerobic step can inhibit the growth of filamentous bacteria, increasing the sludge EPS concentration and promoting the precipitation of activated sludge.

## Introduction

Activated sludge processing is the most widely used biological wastewater treatment technique^1^. However, the common occurrence of sludge bulking severely impacts the normal operation of activated sludge biological wastewater treatment systems, affecting the effluent quality and increasing the time required for the system to recover^2^. There are two primary causes of sludge bulking: the first is the filamentous bulking caused by the excessive proliferation of filamentous bacteria, while the second is the viscous bulking caused by the large amount of extracellular polymeric substances (EPS) produced by zoogloea. The primary cause for the filamentous bulking is the different reactions of microflora to environmental changes, resulting in the disruption of the microflora balance in the sludge system^3^. The excessive proliferation of some filamentous bacteria^4,5^ or filamentous fungi^6^ increases the difficulty of separating the sludge and water. EPS are natural high molecular weight polymers secreted during the growth and metabolism of microorganisms. EPS are primarily composed of polysaccharides and proteins, contain abundant functional groups, and are the primary components of sludge flocs. Therefore, they are closely related to the settleability of sludge flocs^7^. Numerous studies of sludge bulking have been performed and a series of technical approaches and measures have been suggested to prevent and control sludge bulking^8–10^, one of which is the addition of an anaerobic step^11,12^. The setup of the anaerobic step for the anaerobic biological selector can radically inhibit the growth of filamentous bacteria. Moreover, because there is no need to add chemical reagents, it is a sustainable method to inhibit sludge bulking and is therefore widely used in biological wastewater treatment plants^13,14^. Research on setting up the anaerobic step for the anaerobic biological selector to inhibit sludge bulking is primarily focused on theoretical studies^15^ and the apparent inhibition effect^16^. Studies have shown that the setup of the anaerobic step relies on the “selection pressure” resulting from the high load gradient to select zoogloea. Based on the storage selection theory, the organic carbon sources in the influent are largely consumed by various biochemical reactions, such as storage and phosphorus release during the anaerobic step. This will cut the nutritional supplies for the subsequent growth of filamentous bacteria during the aerobic step^17^, inhibiting the growth of filamentous bacteria.

Although research capabilities and technical approaches have improved in recent years, few studies have taken advantage of these advances to explain the inhibition of sludge bulking in the anaerobic step. Therefore, it is particularly important to carry out studies in this area. In the present study, after sludge was bulked in a sequencing batch reactor (SBR), an anaerobic step was added after the influent step to investigate its effect on the settleability of sludge. High-throughput sequencing was used to analyse the changes in the bacterial community composition of the sludge after adding the anaerobic step. In addition, the EPS of the activated sludge was also analysed. This study aims to provide a theoretical basis and support for the prevention and resolution of sludge bulking issues in the design and operation of activated sludge processes in wastewater treatment plants.

## Results

### Pollutant removal

During the entire running cycle, the Chemical Oxygen Demand (COD) effluent concentration of the reactor was 6~47 mg·L^−1^. The sludge bulking stage from day 109 to day 130 was caused by the decreased DO concentration. Because there was not enough DO for nitration, the reaction was incomplete, and the effluent ammonium concentration was 14.90~43.76 mg·L^−1^. In other operation stages, the effluent ammonium concentration was 0.80~4.92 mg·L^−1^, exhibiting an excellent ammonium removal efficiency.

### Variation of sludge settleability

Sludge settleability was evaluated by determining the SVI of activated sludge. When the SVI is greater than 150 mL·g^−1^, the sludge is considered to be bulked. From day 1 to day 118, the SVI of the sludge was 26~123 mL·g^−1^, and the sludge had excellent settleability. From day 119 to day 153, the SVI of sludge was 166~212 mL·g^−1^, and the sludge was in a bulking state. The anaerobic step was added on day 154, and after only 10 operation cycles on day 158 the sludge settleability was restored, with the SVI of the sludge gradually decreasing to 133 mL·g^−1^. Thus, the addition of anaerobic step rapidly inhibited sludge bulking. The average rate at which the SVI of the activated sludge decreased was 8.9 mL·(g·cycle)^−1^. The sludge settleability was restored to normal from day 158 to day 208 with an SVI of 56~133 mL·g^−1^.

### Microscopic examination of sludge

**Figure 1:**
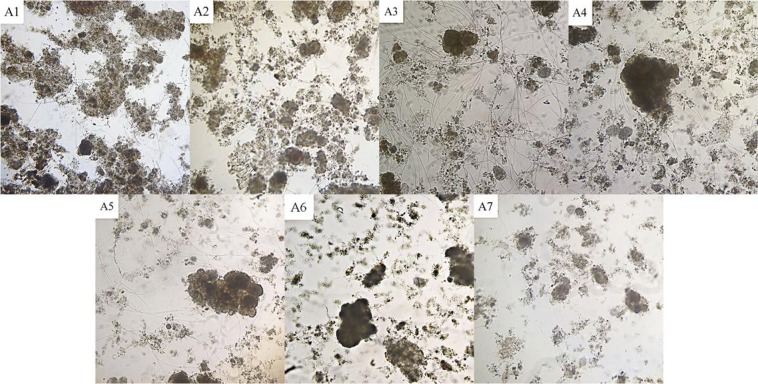
Microscopic examination of sludge (100×).

The sludge samples were examined by optical microscopy. As shown in Fig. 1, in sludge samples A1 and A2 a large number of evenly distributed zoogloea were observed with a very small amount of filamentous bacteria. In the A3 bulked sludge sample, very few zoogloea were observed, but an abundance of filamentous bacteria with long and straight filaments were present. The number of filamentous bacteria gradually decreased in the A4 and A5 sludge samples. For the A6 and A7 samples, in which bulking was restored, almost no filamentous bacteria were observed, and the number of zoogloea gradually increased. The microscopic examinations showed that filamentous bulking was the sludge bulking process occurring in the experiment.

### Analysis of bacterial community of the activated sludge before and after adding an anaerobic step

Table 1 shows that 30,119~43,862 filtered bacterial sequences were obtained that were grouped into 604~673 Operational Taxonomic Units (OTUs), while 30,711~44,718 filtered fungal sequences were obtained that were grouped into 17~44 OTUs. The bacterial coverage index was greater than 0.996, and the fungal coverage index all reached 1.000.

**Table 1:** Bacterial and fungal diversity analysis.

Microbial communit sample		Number of sequences	Number of OTUs	Chao index	Shannon index	Coverage index
Bacteria	A1	37963	627	701	4.93	0.997
	A2	39085	635	735	4.78	0.996
	A3	31573	651	744	4.75	0.996
	A4	30884	612	665	4.80	0.997
	A5	43862	641	698	4.70	0.996
	A6	30119	673	762	4.75	0.996
	A7	41462	604	674	4.44	0.996
Fungi	A1	44718	17	44	1.84	1.000
	A4	30711	44	32	0.56	1.000
	A6	32605	26	28	0.66	1.000
	A7	35830	18	27	1.06	1.000

**Figure 2:**
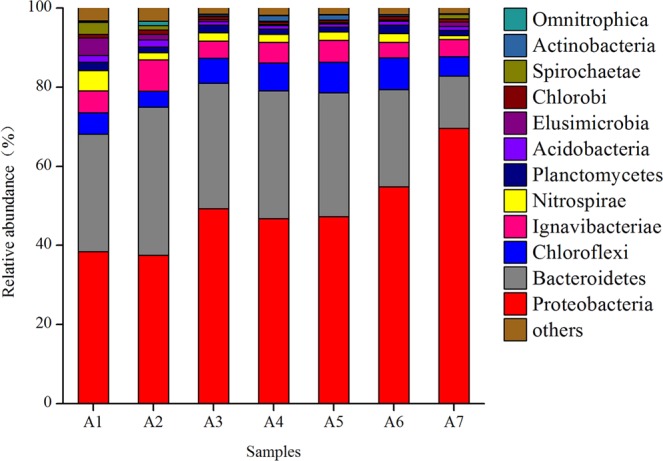
Variation in the relative abundance of bacterial phylum in sludge samples.

All bacterial OTUs belonged to 30 bacterial phyla. In at least one sludge sample, 12 bacterial phyla were present with relative abundances of greater than 1.0%. The relative abundances of bacterial phyla are shown in Fig. 2. The primary bacterial phyla included Proteobacteria (37.5~69.6%), Bacteroidetes (13.2~32.3%), Chloroflexi (5.0~8.0%), Ignavibacteriae (3.9~5.6%), Nitrospirae (1.0~5.1%), Elusimicrobia (0.3~4.4%), Spirochaetae (0.1~3.0%), Actinobacteria (0.02~1.4%) and others, which play key roles in the removal of pollutants in wastewater.

The bacterial diversity of the sludge from A1 to A7 was principally composed of OTUs associated to the phylum Proteobacteria and Bacteroidetes. The phylum Proteobacteria exhibited the greatest relative abundance among all sludge bacterial phyla, with relative abundances in A1 and A2 samples of 38.4 and 37.5%, respectively. The relative abundance of Proteobacteria increased to 49.3% in the A3 bulked sludge sample, and during the restoration of settleability by adding an anaerobic step, the relative abundance of this phylum gradually increased to 69.6% in A7 sample, which exhibited fully restored settleability. The relative abundance of Bacteroidetes, Chloroflexi, Ignavibacteriae and Acidobacteria were not obvious. The relative abundance of Actinobacteria was 0.05 and 0.02% in A1 and A2 samples before bulking, respectively, increasing to 0.4% in A3 the bulked sludge sample and to 1.4% in the A4 sludge sample at the beginning of the settleability restoration. However, for the A7 sample, which had fully restored settleability, the relative abundance decreased to 0.2%. The relative abundances of the phyla Nitrospirae, Elusimicrobia and Spirochaetae were 5.1, 4.4 and 3.0% in the A1 sludge sample, decreasing to 2.1, 0.5 and 0.1% in the A3 bulked sludge sample, respectively. When the anaerobic step was added, the relative abundances of these bacterial phyla slightly increased.

**Figure 3:**
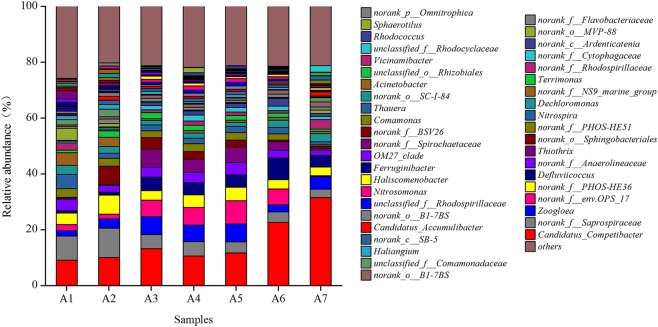
Variation in the relative abundance of bacterial genus in sludge samples.

In total, 342 bacterial genera were observed from sequencing sludge samples. In at least one sludge sample, 14 bacterial genera were present with relative abundances of greater than 1.0%. The relative abundance of bacterial genera is shown in Fig. 3. The primary bacterial genera observed included *Candidatus*_*Competibacter* (9.2~31.4%), *norank_f_Saprospiraceae* (3.0~10.5%), *Zoogloea* (1.8~6.4%), *norank_f_env.OPS_17* (0.2~8.4%), *norank_f_PHOS-HE36* (3.1~6.9%), *Defluviicoccus* (0.9~7.7%), *norank_f_Anaerolineaceae* (1.9~4.6%) and others. *Candidatus*_*Competibacter* had the highest relative abundance among all samples and was the dominant bacterial genus. The relative abundance of *Candidatus*_*Competibacter* in the A1 and A2 sludge samples was 9.2 and 10.1%, and that of *Defluviicoccus* was 0.9 and 1.0%, respectively. The relative abundances of these two genera increased to 13.2 and 4.9% in sample A3 to 22.6 and 7.7% in sample A6 and to 31.4 and 4.1% in sample A7, respectively. *Candidatus*_*Accumulibacter* was present at a relative abundance of 1.4% in the A2 sludge samples, decreased to 6.3% in the A3 bulked sludge sample, then gradually increased after adding the anaerobic step, reaching 1.5% in the A7 sludge sample with fully restored settleability. *Thiothrix* and *norank_o_Sphingobacteriales* exhibited notable changes in relative abundance throughout the experiment. *Thiothrix* was present at a relative abundance of 0.2 and 0.3% in the A1 and A2 sludge samples, respectively, which exhibited excellent settleability at the beginning of the experiment. The relative abundance of *Thiothrix* increased to 6.3% in the A3 bulked sludge sample, then gradually decreased after adding the anaerobic step, reaching 3.0% in the A6 and reaching 0.7% in the A7 sludge sample with fully restored settleability. The relative abundance of *norank_o_Sphingobacteriales* was 0.2% in the A1 sludge sample, increasing to 6.6% in the A2 sludge sample and was 4.3% in the A3 bulked sludge sample. The relative abundance of *norank_o_Sphingobacteriales* gradually decreased after adding the anaerobic step to 0.6 and 0.8% in the A6 and A7 sludge samples, respectively. The relative abundance of *norank_f_Saprospiraceae* was 8.6 and 10.5% in the A1 and A2 sludge samples, respectively, which had excellent settleability at the beginning of the experiment. Because of the impact of sludge bulking, the relative abundance of* norank_f_Saprospiraceae* decreased to 5.0% in the A3 bulked sludge sample and to 3.0% in the A7 sludge sample with fully restored settleability. Due to sludge bulking, the relative abundance of *Nitrospira* decreased from 5.1% in the A1 sludge sample to 2.1% in the A3 bulked sludge sample. This value further decreased to 1.0% in the A7 sludge sample with fully restored settleability. Therefore, the relative abundance was greatly affected by the filamentous sludge bulking.

### Analysis of fungal community of the activated sludge before and after adding an anaerobic step

**Figure 4:**
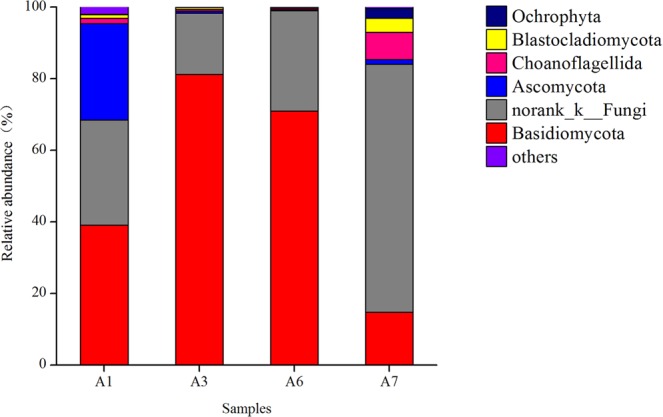
Variation of relative abundance of fungal phylum in sludge samples.

Sixteen fungal phyla were observed from in the sludge samples. In at least one sludge sample, 6 fungal phyla were present with relative abundances of greater than 1.0%. The relative abundances of fungal phyla are shown in Fig. 4. The primary fungal phyla included Basidiomycota (14.7~81.2%), norank_k_—_Fungi (17.1~69.3%), Ascomycota (0.3~26.9%) and others. Their relative abundances of these phyla in the A1 sludge sample were 39.1, 29.5 and 26.9%, respectively, thus exhibiting a relatively even distribution. However, the relative abundances of these phyla exhibited significant changes after sludge bulking. In the A3 bulked sludge sample, the relative abundance of Basidiomycota increased to 81.2% but was only 14.7% in the A7 sludge sample with excellent settleability. The relative abundance of Ascomycota was 26.3% in the A1 sludge sample, which exhibited excellent settleability at the beginning of the experiment, decreasing to below 1.0% after sludge bulking, indicating that the relative abundance was greatly affected by sludge bulking. The relative abundance of norank_k_—_Fungi was 29.5% in the A1 sludge sample, decreasing to 17.1% in the bulked sludge and then gradually increased after adding the anaerobic step. For example, the relative abundance of this phylum was 28.0 and 69.3% in the A6 and A7 sludge samples, respectively.

**Figure 5:**
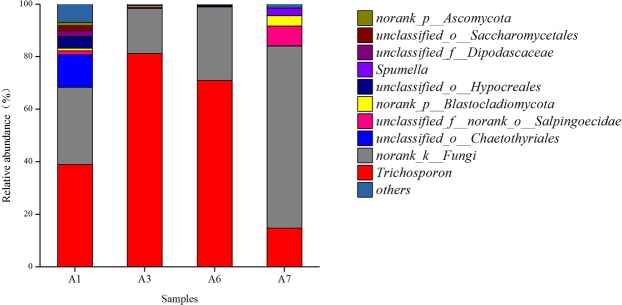
Variation of relative abundance of fungal genus in sludge samples.

Thirty-five fungal genera were observed in the sludge samples. In at least one sludge sample, 10 fungal genera were present with relative abundances of greater than 1.0%. The relative abundances of fungal genera are shown in Fig. 5. The relative abundance of *Trichosporon* was 38.9% in the A1 sludge sample with excellent settleability at the beginning of the experiment, increasing to 81.2% in the A3 bulked sludge sample and decreasing to 14.7% in the A7 sample after adding the anaerobic step. The relative abundance of *norank_k_Fungi decreased* from 29.5% in the A1 sludge sample to 17.1% in the A3 sample, gradually increasing to 69.3% in the sample A7. However, the relative abundances of *norank_o_Salpingoecidae*, *norank_p_Blastocladiomycota* and *Spumella* were 1.5, 0.9 and 0.1% in the A1 sludge sample, respectively. These three groups were lower than 1.0% in the A3 bulked sludge and the A6 sludge samples, increasing to 7.6, 3.9, and 2.8% in the A7 sludge sample with fully restored settleability, respectively. Therefore, *norank_o_Salpingoecidae*, *norank_p_Blastocladiomycota* and *Spumella* are favourable for the improvement of sludge settleability.

### Relationship between the EPS concentration and settleability of the activated sludge before and after adding an anaerobic step

The sludge EPS concentration in the reactor was measured when the operation from day 109 to 171 of the experiment. Because the measured humic acid and DNA concentrations were low during the experiment, the total EPS was represented by the sum of polysaccharides and protein concentrations. The protein concentration was 26.8~74.8 mg·(gMLSS)^−1^, accounting for 59.2~92.6% of the total EPS, while the polysaccharide concentration was 3.1~32.9 mg·(gMLSS)^−1^, accounting for 7.4~40.8% of the total EPS. Thus, proteins were the primary component of EPS in this study.

## Discussion

Filamentous sludge bulking occurred to the activated sludge. By adding a 30 min anaerobic step before the aerobic step, the amount of sludge zoogloea was increased while that of filamentous bacteria decreased. The average rate of the SVI decrease of activated sludge was 8.9 mL·(g·cycle)^−1^. Only 10 running cycles were required to rapidly and effectively inhibit sludge bulking.

Microbial community analysis provided crucial information to understand adding an anaerobic step can rapidly inhibit sludge bulking. High-throughput sequencing was used to analyse the bacterial community composition of the activated sludge in the SBR reactor. The coverage index was greater than 0.996, and the fungal coverage index all reached 1.000, indicating that the depth of the sequence was sufficient. The higher the Chao index value, the richer the species richness, while the higher the Shannon index, the higher the diversity of the communities^18^. There was little change in the bacterial Chao and Shannon indices for each sample. During the sludge bulking process, the richness of the bacterial community gradually increased, but the diversity gradually decreased. After adding the anaerobic step, the bacterial community richness first decreased and then gradually increased during the sludge settleability restoration process, while the bacterial diversity initially increased and then gradually decreased. The fungal richness and diversity in sample A1 was higher than those of the A3, A6 and A7 samples. Sludge bulking influenced the fungal community in that the fungal richness and diversity decreased due to sludge bulking. After adding the anaerobic step, the fungal richness decreased, while the diversity gradually increased.

The phylum Proteobacteria exhibited the greatest relative abundance among all sludge bacterial phyla, which is consistent with the results of the bacterial communities in soil^19–21^ and activated sludge^22,23^. Proteobacteria have wide distribution and high diversity^24^, and their primary function during wastewater treatment is to remove organic pollutants, nitrogen and phosphorus^25^. Because of the eurytopic nature of the phylum Proteobacteria, its relative abundance was increased after adding the anaerobic step. Bacteroidetes, Chloroflexi, and Acidobacteria were also often observed in activated sludge^24,26^, Bacteroidetes have strong metabolic capacity for complex organics, proteins and lipids, and can decompose complex macromolecules into simple compounds, which play an important role in the ecosystem^27^. Chloroflexi is mostly filamentous bacteria, and exists in the form of flocs skeleton in flocculent sludge clump inside body, play a role of sludge flocculation, at the same time with macromolecular organic matter degradation ability and has good biological phosphorus removal effect^28^. Firmicutes may be related to the degradation of COD and refractory macromolecules^22^. Research has shown that the excessive growth of Actinobacteria can induce filamentous sludge bulking^29^. The relative abundances of the phyla Nitrospirae, Elusimicrobia and Spirochaetae decreased in the bulked sludge sample, indicating that the excessive proliferation of filamentous bacteria inhibited the growth of these microorganisms.

*Thiothrix* and *norank_o_Sphingobacteriales* exhibited notable changes in relative abundance throughout the experiment, both *Thiothrix* and* norank_o_Sphingobacteriales* showed a noticeable increase. The excessive proliferation of filamentous *Thiothrix* can cause sludge bulking^30^.These observations indicated that the excessive proliferation of these two bacteria is the primary reason for the observed filamentous sludge bulking in the experiments.

The excessive proliferation of *Thiothrix* and *norank_o_Sphingobacteriales* in the bacterial genera resulted in sludge bulking. After adding the anaerobic step as an anaerobic selector, the growth of *Thiothrix* and *norank_o_Sphingobacteriales* were inhibited. The sludge bulking was rapidly and effectively inhibited.

Similar results were found in studies by Donkin and Nielsen. The presence of an anaerobic tank ahead of a completely mixed aerobic reactor as an anaerobic selector was to limit/suppress *Thiothrix*-caused bulking in dairy wastewater treatment plants^31^. Nielsen’s^32^ research shows that *Thiothrix* remained physiologically active under prolonged anaerobic conditions, but it seemed that the growth of *Thiothrix* was limited in such environments with an anaerobic selector ahead of a completely mixed aerobic conditions. In addition, other research shows that^12^ adding the anaerobic step as an anaerobic selector generally showed good performances in controlling filamentous bacteria when treating both municipal and industrial wastewaters, namely when bulking occurred following Type 0041 overgrowth. Constantinos *et al*.^33^ proved that under the conditions prevailing in anaerobic selector tank, the growth of *M*. *parvicella* was limited.

In activated sludge, both glycogen accumulating organi (GAO) and phosphorusaccumulatingorganism (PAO) were reported to help structuring the biomass into dense and stable aggregates^34,35^ and help increasing the settleability of sludge. Most common GAO reported were the Gammaproteobacterium *Candidatus*_*Competibacter* and the Alphaproteobacterium *Defluviicoccus*^36,37^, most common PAO were the Betaproteobacterium *Candidatus*_*Accumulibacter*. In the anaerobic step, these two glycogen-accumulating bacteria *Candidatus*_*Competibacter*, *Defluviicoccus* and *Candidatus*_*Accumulibacter* adsorb large amounts of organic substrates, such as volatile fatty acids, converting them to intracellular polymers such as polyhydroxyalkanoates for storage and inhibiting the growth of filamentous bacteria. This is also beneficial for the formation of high-density sludge particles, further increasing the settleability of sludge^38^. The filamentous bulking was solved after adding the anaerobic step and a significant population of *Candidatus*_*Competibacter*, *Defluviicoccus* and *Candidatus*_*Accumulibacter* was established.

Although sludge bulking caused a decrease in the relative abundance of *Nitrospira*, members of which played an important role in the biodenitrification process, the effluent ammonia nitrogen concentration did not increase. This result occurred because the DO concentration in the mixed solution was greater than 2.0 mg·L^−1^, which improved the activity of *Nitrospira*^39,40^ and its ammonia nitrogen oxidation capability.

High-throughput sequencing was used to analyse the fungi community composition of the activated sludge in the SBR reactor also. The primary fungal phyla included Basidiomycota, norank_k_—_Fungi and Ascomycota, which are typically present in the activated sludge of urban wastewater treatment plants^41^. In the bulked sludge sample, the relative abundance of Basidiomycota increased and decreased in sludge sample with excellent settleability after adding an anaerobic step before the aerobic step. These results indicate that the excessive proliferation of Basidiomycota is unfavourable to the settlement of sludge, while the addition of the anaerobic step can effectively inhibit the proliferation of this fungal phylum.

The relative abundance of norank_k_—_Fungi was increased after adding an anaerobic step before the aerobic step, indicating that the improvement in the relative abundance of norank_k_—_Fungi in sludge is favourable to the settleability of sludge, and the addition of an anaerobic step is favourable for the growth of norank_k_—_Fungi and can promote the growth and proliferation of norank_k_—_Fungi, thus improving the settleability of sludge.

The relative abundance of *Trichosporon* was 38.9% in the sludge sample with excellent settleability at the beginning of the experiment, increasing to 81.2% in the bulked sludge sample and decreasing to 14.7% in the sample after adding the anaerobic step. *Trichosporon* can induce sludge bulking^6^, the excessive proliferation of *Trichosporon* resulted in sludge bulking in the SBR reactor in this study. After adding the anaerobic step, the growth of these bacteria and fungi were inhibited.

**Figure 6:**
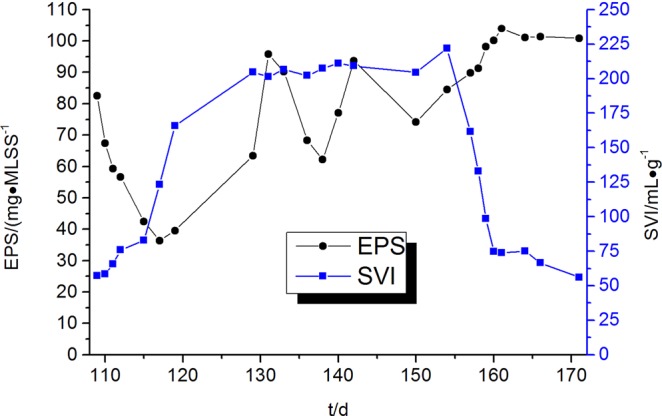
Variation of EPS concentration and SVI value in the sludge samples.

Akkache *et al*.^42^ showed that the presence of EPS improved the settleability of activated sludge. However, other studies^43,44^ concluded that the EPS concentration is positively correlated to the sludge SVI values and is thus unfavourable for the settleability of sludge. The variation in the EPS concentration and SVI values in the sludge in the experiment are shown in Fig. 6. From day 117 to day 131, the polysaccharide concentration in the EPS of the sludge rapidly increased from 3.61 to 25.86 mg·(gMLSS)^−1^. The rapid increase in the polysaccharide concentration causes an increase of electrophoretic mobility and an increase of the repulsive force between the sludge flocs. As a result, the settleability of sludge was worsened^45^, and the SVI value increased from 123 to 201 mL·g^−1^. The change in the bacterial community composition in the activated sludge from day 154 to day 161 after adding the anaerobic step resulted in a change in the EPS concentration^46^, which increased from 84.4 to 104.0 mg·(gMLSS)^−1^, and the concentration of proteins secreted by microorganisms rapidly increased from 60.2 to 74.3 mg·(gMLSS)^−1^. The increase in the protein concentration decreases the effective critical potential on the surface of microorganisms and promotes bioflocculation^47^. The sludge Settling Velocity (SV) rapidly decreased from 92 to 39%, and the sludge SVI value decreased from 222 to 74 mL·g^−1^. The increase in the polysaccharide concentration in the EPS of sludge is unfavourable to the settlement of activated sludge. The change in the bacterial community composition after adding the anaerobic step resulted in an increase of protein concentration in the EPS in the sludge, promoting the settlement of activated sludge. The growth and proliferation of filamentous bacteria was inhibited after adding the anaerobic step. The bacterial and fungal community compositions in the sludge exhibited significant changes. The change of dominant bacteria and fungi resulted in the increase of EPS concentration in the sludge and promoted the restoration of the settleability of sludge.

## Materials and Methods

### Experimental equipment and operation mode

SBR reactor with an effective volume of 11 L were used as the experimental equipment, running for 2 cycles every day with a 10 min water influent step, a 360 min aerobic step, a 60 min precipitation step, a 10 min drainage step with a drainage ratio of 38.5%, and a 290 min standing step. After adding the anaerobic step, the equipment was run with a water 10 min water influent step, a 30 min anaerobic step, a 360 min aerobic step, a 60 min precipitation step, a 10 min drainage step, and a 260 min standing step.

### Seed sludge and experimental water

The seed sludge was taken at the end of the aerobic step of the oxidation ditch process in a wastewater treatment plant in the Changji city of Xinjiang.The MLSS of the seed sludge was 4,512 mg·L^−1^ and the SVI was 202 mL·g^−1^. The experimental water used was synthetic water that simulated domestic wastewater. The primary components of the synthetic water included CH_3_COONa·3H_2_O, NH_4_Cl, KH_2_PO_4_, MgSO_4_·7H_2_O and CaCl_2_, while the trace components included FeSO_4_·7H_2_O, CuSO_4_·5H_2_O, H_2_BO_4_, MnSO_4_·H_2_O, NaMo_4_·2H_2_O, ZnCl·7H_2_O and CoCl·6H_2_O. The COD concentration was 520 mg·L^−1^, the Total Nitrogen (TN) concentration was 52 mg·L^−1^ and the Total Phosphorus (TP) concentration was 5.2 mg·L^−1^.

### Experimental process and sampling

Bulked sludge was used as the seed sludge used in this study. After excellent settleability was maintained, the experiment was initiated and lasted for 208 days. The experimental process was as follows. From day 1 to day 108, the operation status remained unchanged, with a (DO) concentration of 1.09~4.98 mg·L^−1^. From day 109 to day 130, while other parameters remained constant, the DO concentration decreased to 0.20~0.65 mg·L^−1^, resulting in the gradual bulking of sludge. From day 131 to day 153, the aeration rate was restored such that the DO concentration was 2.19~3.50 mg·L^−1^, although the settleability of sludge was not restored, even with sufficient DO. From day 154 to day 208, after water was added to the reactor, a 30 min anaerobic step was added prior to the aerobic step.

The sampling and numbering scheme is shown in Table 2. Sludge samples A1 and A2 had excellent settleability at the beginning of the experiment; sludge sample A3 was taken when the sludge bulking occurred; sludge samples A4, A5 and A6 were taken from the stage when the settleability of sludge was restored after adding the anaerobic step; and sludge sample A7 had fully restored settleability after the addition of an anaerobic step.

**Table 2:** Sampling time, sludge settling performance, pH, dissolved oxygen and water temperature of the operation.

Sample	Days of operation (d)	SV (%)	SVI (mL·g^−1^)	pH	DO (mg·L^−1^)	Water temperature (°C)
A1	3	29	52	8.4	2.32	21
A2	64	69	98	8.2	3.24	19
A3	122	90	212	8.3	0.35	20
A4	158	72	133	8.2	2.72	22
A5	160	44	75	8.5	3.14	22
A6	166	32	67	8.4	3.25	24
A7	171	26	56	8.3	2.56	28

### Analytical methods for water quality and sludge monitoring

Ammonium and COD were analyzed according to standard procedures. DO was measured with a portable DO analyser; pH was measured with a pH test pen; and the water temperature was measured with a mercury thermometer. Sludge volume index (SVI) were determined by reading the volume of the settled bed in a column after 30 min settling and calculated from the dry weight in (MLSS). Microscopic observations were performed with a photonic microscope. The morphology of filaments and flocs was evaluated on a day-to-day basis.

### EPS extraction methods and component analysis methods

EPS was extracted through an ethylene diamine tetraacetic acid (EDTA) method, the activated sludge was harvested by centrifugation at 5600 rpm for 10 min at 4°C, the collected activated sludge was re-suspended in fresh sterile and harvested by centrifugation at 5500 rpm for 20 min at 4°C, and the collected activated sludge was re-suspended in fresh sterile and 2%EDTA (1:1) at 4 °C for 3 h, then harvested by centrifugation at 12300 rpm for 20 min at 4°C.Extractant residues in the solution were removed by the dialysis membrane filtration in the subsequent treatment. The supernatants were collected as EPS solution and stored at 4 °C or freeze-dried for further analysis.The carbohydrate content in EPS was measured by the anthrone method^48^ using glucose as the standard. The contents of protein in EPS were measured by the modified Lowry method^49^ using bovine serum albumin as the respective standards.

### DNA extraction, PCR amplification and Illumina sequencing

Microbial DNA was extracted from sludge samples collected from the SBR reactor using the E.Z.N.A.® soil DNA Kit (Omega Bio-tek, Norcross, GA, U.S.). The final DNA concentration and purification were determined by NanoDrop 2000 UV-vis spectrophotometer (Thermo Scientific, Wilmington, USA), whereas DNA quality was checked by 1% agarose gel electrophoresis. The V4-V5 hypervariable regions of the bacteria 16 S r RNA gene was amplified with primers 515 F (5ʹ-GTGCCAGCMGCCGCGG-3ʹ) and 907 R (5ʹ-CCGTCAATTCMTTTRAGTTT-3ʹ), the fungal 16 S r RNA gene was amplified with primers 0817 F (5′-TTAGCATGGAATAATRRAATAGGA-3′) and 1196 R (5′-TCTGGACCTGGTGAGTTTCC-3′) by thermocycler PCR system (GeneAmp 9700, ABI, USA). The PCR reactions were conducted using the following program: 3 min of denaturation at 95 °C, 27cycles of 30 s at 95 °C, 30 s for annealing at 55 °C, and 45 s for elongation at 72 °C, and a final extension at 72 °C for 10 min. PCR reactions were performed in triplicates of 20 μL mixture containing 4 μL of 5 × FastPfu Buffer, 2 μL of 2.5 mM dNTPs, 0.8 μL of each primer (5 μM), 0.4 μL of FastPfu Polymerase and 10 ng of template DNA. The PCR products were extracted from a 2% agarose gel and further purified using the AxyPrep DNA Gel Extraction Kit (Axygen Biosciences, Union City, CA, USA). Then, the products were quantified using QuantiFluor™-ST (Promega, USA).

Purified amplicons were pooled in equimolar and paired-end sequenced (2 × 300) on an Illumina MiSeq platform (Illumina, San Diego, USA) according to the standard protocols of Majorbio Bio-Pharm Technology Co. Ltd. (Shanghai, China). The raw reads were deposited into the NCBI Sequence Read Archive (SRA) database (Accession Number: SRP127708).

### Data analysis

Data analysis was conducted using the i-sanger platform (http://www.i-sanger.com/) provided by Majorbio Bio-Pharm Technology Co. Ltd. (Shanghai, China). The microbial phylotype richness levels were calculated using the Chao estimator, the Shannon diversity index. The Chao estimator, the Shannon diversity index and the coverage percentage were also calculated by the Mothur program version v.1.30.1 (http://www.mothur.org/wiki/Schloss_SOP#Alpha_diversity). These analyses were performed using the R Programming Language software.
